# Treatment of Lupus Exedens

**Published:** 1871-09

**Authors:** E. Andrews

**Affiliations:** Prof. of Surgery in Chicago Medical College; Chicago, No. 6, Sixteenth Street


### TREATMENT OF LUPUS EXEDENS.

BY E. ANDREWS, M.D. Prof. of Surgery in Chicago Medical College.

The vagueness of diagnosis which hangs like a cloud about the subject of lupus, prevents the development of well-defined ideas of its treatment. The London Hospital for skin diseases employs the actual cautery in most cases, and many surgeons in private practice do the same with excellent effect. The horror of patients at the idea of the hot iron renders it, however, a rather inconvenient remedy in American practice. The German surgeons also complain of the extent of the cicatrices produced in this way. Hebra prefers to gouge out all the tissue which can be reduced to a pulp by the action of the finger nails. R. Volhmann, of Halle, takes the following course:—with an instrument like a small spoon, he first scoops away all the tissue which will yield to its scraping action, forcibly applied, then with a tenotome, or other small knife, he makes innumerable minute slashes and stabs into all the affected vascular tissue around, cutting until he reduces it to a sort of mince-meat, without, however, destroying the vitality of any of it. The ulcers then begin to heal, and the contraction of the multitudinous small cicatrices reduces the affected surrounding tissue to a nearly natural condition. I have recently tried this plan with excellent effect in Mercy Hospital. The patient had lost the septum narium and part of the border of the nose, and of the upper lip. I removed all the diseased parts which would yield to a vigorous scraping action, and then slashed and stabbed all the red tissue in the vicinity. An immediate improvement began to take place, and in about four weeks the parts were healed. The tip of the nose, which had been drawn down, closing the orifice of the nares, and rendering respiration by that passage impossible, was supported by a gutta percha tube, and, as the cicatrix grew firmer, showed its power to maintain its position without further help. On the whole the results were so favorable as to give great encouragement to the further trial of Volhmann's method.

CHICAGO, *No. 6, Sixteenth Street.*

